# Geographic Progression of Infant Respiratory Syncytial Virus Associated Bronchiolitis Across the United States Before and Since the Onset of COVID‐19: Results From Four Health Systems, 2015–2023

**DOI:** 10.1111/irv.13298

**Published:** 2024-05-15

**Authors:** Adam Z. Blatt, Mina Suh, Emmanuel B. Walter, Charles T. Wood, Claudia Espinosa, Maria E. Enriquez‐Bruce, Joseph Domachowske, Danielle Daniels, Sonia Budhecha, Amanda Elliott, Zachary Wolf, Emory B. Waddell, Naimisha Movva, Heidi Reichert, Jon P. Fryzek, Christopher B. Nelson

**Affiliations:** ^1^ Pediatric Infectious Diseases Duke University Health System Durham North Carolina USA; ^2^ EpidStrategies, A Division of ToxStrategies Rockville Maryland USA; ^3^ Duke Human Vaccine Institute Durham North Carolina USA; ^4^ General Pediatrics and Adolescent Health Duke University Health System Durham North Carolina USA; ^5^ Pediatric Infectious Diseases University of South Florida Health Tampa Florida USA; ^6^ Pediatric Infectious Diseases State University of New York Upstate Medical University Syracuse New York USA; ^7^ Pediatric Pulmonology Renown Health Reno Nevada USA; ^8^ University of Nevada, Reno School of Medicine Nevada Reno USA; ^9^ Clinetic Durham North Carolina USA; ^10^ Sanofi Swiftwater Pennsylvania USA

**Keywords:** epidemiology, respiratory syncytial virus, seasonality, surveillance

## Abstract

**Background:**

Respiratory syncytial virus (RSV) is a substantial cause of infant morbidity and mortality due to seasonal peaks of bronchiolitis across the United States. Clinical and viral surveillance plays a pivotal role in helping hospital systems prepare for expected surges in RSV bronchiolitis. Existing surveillance efforts have shown a geographic pattern of RSV positivity across the United States, with cases typically starting in the southeast and spreading north and west. Public health measures implemented due to the COVID‐19 pandemic disrupted viral transmission across the nation and altered the expected seasonality of RSV. The impact of these changes on the geographic progression of infant RSV bronchiolitis across the United States has not been described.

**Methods:**

Here, we used clinical and viral surveillance data from four health care systems located in different regions of the United States to describe the geographic progression of infant RSV bronchiolitis across the country from 2015 to 2023.

**Results:**

Prior to widespread circulation of SARS‐CoV‐2, infant RSV bronchiolitis followed an established geographic pattern associated with seasonal epidemics originating in Florida and spreading north (North Carolina and New York) and later westward (Nevada). Although public health and social measures implemented during the COVID‐19 pandemic disrupted the seasonality of RSV disease, infant RSV bronchiolitis epidemics progressed across the nation in a pattern identical to the prepandemic era.

**Conclusions:**

Our findings highlight the importance of ongoing clinical and viral surveillance to optimally track the onset of RSV epidemics and allow health care systems to prepare for expected RSV bronchiolitis surges.

## 1 Introduction

Respiratory syncytial virus (RSV) is among the most common causes of lower respiratory tract infections in children, worldwide [1]. RSV bronchiolitis is also the leading cause of hospitalization among United States (US) infants and accounts for nearly 10% of all hospitalizations each year in this age group [2]. Two preventative agents targeting the RSV preF protein were recently approved for the prevention of RSV specifically among infants: an immunization for pregnant people and nirsevimab, a long‐acting monoclonal antibody [3, 4, 5]. The Centers for Disease Control (CDC) recommends maternal immunization during weeks 32 through 36 of pregnancy, administered September through January, or the long‐acting monoclonal antibody for infants younger than 8 months of age who were born during or are entering their first RSV season [3, 4, 5]. As the guidance for administration of both preventative agents is tightly linked with RSV seasonality, it is essential for health systems to be able to accurately predict the onset of local RSV epidemics.

RSV has well‐described and predictable seasonality in children less than 5 years of age in the US, with cases typically peaking during the winter months each year [2, 6, 7, 8]. Regional variability in the precise timing of these epidemics has also been well characterized [7, 8], although public health and social measures (PHSMs) implemented due to the coronavirus disease 2019 (COVID‐19) pandemic significantly disrupted the expected pattern of respiratory virus transmission across the continental US [6, 9, 10]. While the overall pattern of RSV epidemiology is approaching prepandemic seasonality [10], the effects of this disruption on the regional timing of RSV bronchiolitis epidemics specifically among infants remain unknown.

Utilizing a previously described methodology to define RSV epidemics [7], here, we describe the geographic progression of RSV bronchiolitis among infants across four health care systems located in different US regions, both pre– and post–COVID‐19 pandemic.

## 2 Methods

### 2.1 Study Design and Data Source

Electronic health record (EHR) data were obtained from four health care systems: State University of New York (SUNY) Health System in Syracuse, NY; Duke University Health System (Duke) in Durham, NC; Renown Children's Hospital (Renown) in Reno, NV; and Tampa General Hospital/University of South Florida (TGH/USF) in Tampa, FL. A central Institutional Review Board (IRB) protocol (IRB# 20215411) was approved by each institution to develop the Clinetic NOWcasting platform for real‐time EHR analysis. The ICD‐10 (International Classification of Diseases) codes used to identify encounters for bronchiolitis and RSV bronchiolitis among infants <12 months old were described previously [6]. EHR data were pulled from all medical settings within the health care system, including inpatient, outpatient, intensive care unit (ICU), and the emergency department (ED). Infants were classified as having “RSV bronchiolitis” if they had a positive RSV test (antigen and/or PCR) and an encounter with one of the corresponding ICD‐10 codes (B97.4, J12.1, J20.5, J21.0, J21.8, or J21.9). Infants with any of these same ICD‐10 codes without a documented positive RSV test were classified as having “bronchiolitis” to account for infants who did not undergo RSV testing and/or infants with bronchiolitis encounters due to other viruses. The Clinetic NOWcasting platform began collecting data in Epi week 40 (October 4) of 2015 and was analyzed through Epi week 26 (June 25) of 2023 for this manuscript.

### 2.2 Defining Infant RSV Bronchiolitis Epidemics

Infant RSV bronchiolitis epidemics were defined using previously described methodology that identifies the epidemiological (Epi) weeks [3] that contribute to 75% of the total cases in an RSV season, with the season being defined as Epi week 27 through Epi week 26 of the subsequent year [7]. Only medically attended infants < 12 months old classified as having RSV bronchiolitis (see above) were used to define RSV bronchiolitis epidemics. Since our data started at Epi week 40 in 2015 and we could not determine the effects that the preceding weeks would have on defining that season's infant RSV bronchiolitis epidemic, the 2015–2016 season was excluded from this analysis. The data were analyzed through Epi week 26 in 2023.

The same methodology employed to define seasonal RSV bronchiolitis epidemics among infants was also used to define all‐cause bronchiolitis epidemics at each health care site, to assess the influence of RSV on the timing and duration of all bronchiolitis encounters among infants < 12 months old.

### 2.3 Data Analysis

Descriptive statistics were obtained, and figures were developed, using the Clinetic NOWcasting platform, Microsoft Excel version 16.7, and RStudio version 1.2.1335.

## 3 Results

### 3.1 Patterns of Bronchiolitis Encounters at Four Health Care Sites

**FIGURE 1:**
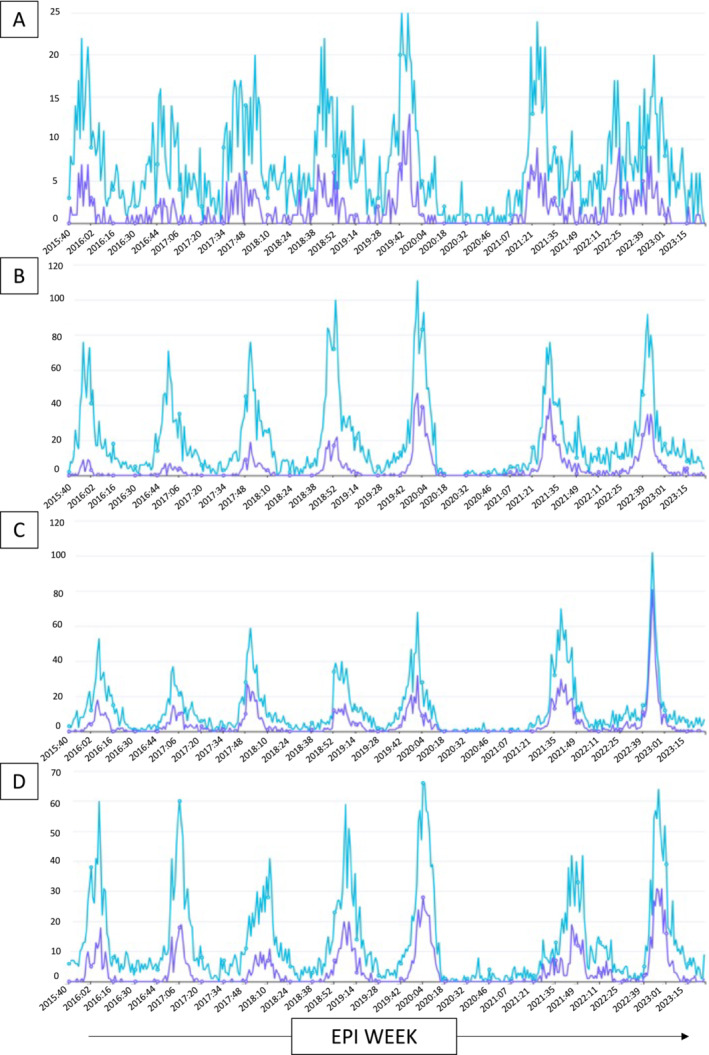
Medically attended bronchiolitis surveillance trends among infants across four regional health care systems. The total number of encounters for bronchiolitis and RSV bronchiolitis among infants < 12 months old for each Epi week are represented by the blue and purple lines, respectively, for each health care system: (A) TGH/USF (Tampa General Hospital and University of South Florida Health in Tampa, FL); (B) Duke (Duke University Health System in Durham, NC); (C) SUNY (State University of New York Upstate Medical University Health System in Syracuse, NY); and (D) Renown (Renown Regional Medical Center Health System in Reno, NV).

**FIGURE 2:**
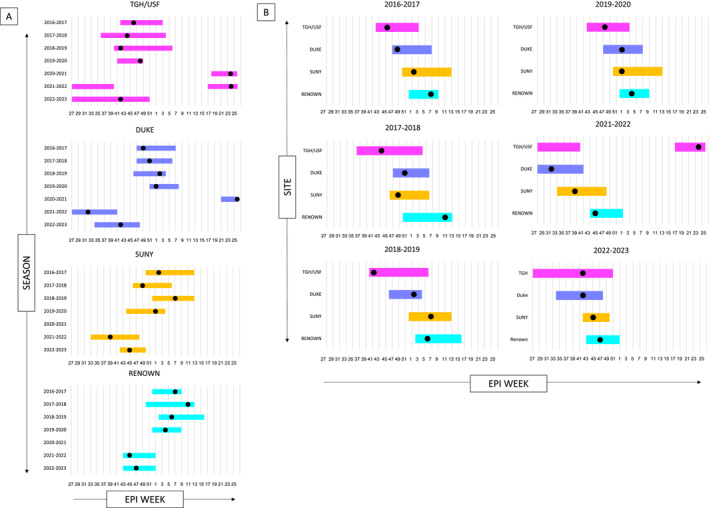
Annual variation in medically attended RSV bronchiolitis epidemics among infants at each health care site. Each bar represents contiguous Epi weeks that comprise 75% of a health care system's total RSV bronchiolitis encounters during a particular RSV season (defined as Epi week 27 through Epi week 26 of the subsequent year). The black dot represents the week within the epidemic that had the peak number of cases. Two bars are shown at TGH/USF for the 2021–2022 season due to the onset of RSV cases prior to Epi week 27 in 2021. (A) depicts epidemics by health care site; (B) depicts epidemics by season. The 2020–2021 season was excluded from (B) due to the lack of an RSV bronchiolitis epidemic at SUNY and Renown, as a result of COVID‐19. TGH/USF (Tampa General Hospital and University of Florida Health in Tampa, FL); Duke (Duke University Health System in Durham, NC); SUNY (State University of New York Upstate Medical University Health System in Syracuse, NY); Renown, Renown Regional Medical Center Health System in Reno, NV.

Prior to the COVID‐19 pandemic, encounters for bronchiolitis among infants <12 months old followed predictable seasonal trends at each health care site with minimal to no inter‐seasonal disease between each annual outbreak. Encounters for RSV bronchiolitis paralleled the general pattern for bronchiolitis encounters among infants (Figure 1). The COVID‐19 pandemic disrupted seasonality for all causes of bronchiolitis, including RSV, across all four health care sites. There was a near complete absence of bronchiolitis diagnoses among infants during the 2020–2021 season, but this was immediately followed by inter‐seasonal disease at all sites (Figures 1 and 2). During the 2021–2022 season, the peak number of cases at each health care site occurred between 2 and 4 months earlier than prepandemic averages. Following the early season disease in 2021–2022, each site also experienced a low level of inter‐seasonal bronchiolitis that extended from the 2021–2022 seasonal peak to the 2022–2023 peak (Figure 1). The 2022–2023 bronchiolitis season also peaked early at all sites, although the peaks shifted closer to established prepandemic patterns.

### 3.2 Geographic Variation in Timing of Infant RSV Bronchiolitis Epidemics Pre–COVID‐19

A general geographic trend in the timing of epidemics was observed (Figure 2), with TGH/USF consistently being the first site to experience RSV bronchiolitis epidemics pre–COVID‐19. The onset of RSV bronchiolitis epidemics at TGH/USF ranged from Epi week 36 to 42 (September–October) and had a median onset at Epi week 40.5. Duke was typically the next site to experience its seasonal RSV bronchiolitis epidemic, although year‐to‐year variability with SUNY was observed prior to COVID‐19. The onset of RSV bronchiolitis epidemics at Duke ranged from Epi weeks 46 to 51 (November–December) with a median onset of Epi week 47, while the onset of RSV bronchiolitis epidemics at SUNY ranged from Epi weeks 44–52 (November–December) with a median onset of Epi week 48. Finally, Renown was consistently the last site to experience its RSV bronchiolitis epidemic with onsets ranging from Epi week 50 to Epi week 2 of the subsequent year (December–January) and a median onset of Epi week 52. The peak weekly case load within the seasonal RSV bronchiolitis epidemic reflected this general geographic trend. Median peaks for each site were as follows: TGH/USF (Epi week 45), Duke (Epi week 52), SUNY (Epi week 1.5), and Renown (Epi week 6.5) (Figure 2).

As an alternative analysis, we determined the timing of the peak number of cases within a rolling 8‐week period at each site to better account for weekly variation (Figure S1). Between the 2015–2016 and 2019–2020 RSV seasons, there was greater variation in the timing of 8‐week RSV bronchiolitis peaks at TGH/USF and SUNY compared to Duke and Renown. The 8‐week peaks at TGH/USF started as early as Epi week 40 and ended as late as Epi week 3 of the subsequent year (October–January). Similarly, the 8‐week peaks at SUNY began as early as Epi week 46 but extended as late as Epi week 12 of the subsequent year (November–March). Eight‐week peaks at Duke consistently occurred between Epi weeks 47 and Epi week 3 of the subsequent year (November–January), with only the 2019–2020 8‐week peak starting slightly later at Epi week 51. Renown also had very consistent 8‐week peaks, as each pre–COVID‐19 epidemic peaked within the same 11‐week period (Epi weeks 1–11, January–March).

### 3.3 Geographic Variation in Timing of Infant RSV Bronchiolitis Epidemics Post–COVID‐19

Despite the shift to early season disease seen in the 2021–2022 and 2022–2023 seasons, the geographic trend in the timing of RSV bronchiolitis epidemics across the four health care sites was preserved (Figure 2). At TGH/USF, RSV bronchiolitis epidemics began in Epi week 18 (May 2) in 2021 and Epi week 17 (April 24) in 2022 with peaks occurring at Epi week 24 (June 12 and 13, respectively) both years, reflecting the early start to the 2021 and 2022 RSV epidemics at this site. Due to the manner in which RSV seasons were defined (as beginning in Epi week 27), another peak was detected at TGH/USF during the 2022–2023 season at Epi week 42 (October 16).

Post–COVID‐19 RSV bronchiolitis epidemics at Duke began in Epi weeks 27 (July 4) and 34 (August 21) and peaked in Epi weeks 32 (August 8) and 42 (October 16) in 2021–2022 and 2022–2023, respectively. SUNY experienced the onset of its post–COVID‐19 epidemics shortly after the RSV bronchiolitis peaks at Duke, with the seasons beginning in Epi week 33 (August 15) in 2021–2022 and Epi week 42 (October 16) in 2022–2023. Weekly cases peaked in Epi weeks 39 (September 26) and 45 (November 6). Again, Renown was the last site to experience the onset and peaks of RSV bronchiolitis epidemics, with epidemics beginning in Epi week 43 (October 24 and 23, respectively) in both post–COVID‐19 seasons and peaking at Epi weeks 45 (November 7) in 2021–2022 and 47 (November 20) in 2022–2023.

### 3.4 Geographic Variation in Duration of Infant RSV Bronchiolitis Epidemics

In addition to geographic variation in the timing of RSV bronchiolitis epidemics among infants, there was also mild variation in the duration of the epidemics at each site (Figure 2). TGH/USF had the longest RSV bronchiolitis epidemics, ranging from 8 to 20 weeks with a median duration of 15.5 weeks pre–COVID‐19. Following the onset of COVID‐19, the RSV bronchiolitis epidemics were extremely drawn out with epidemics starting in early 2021 (Epi week 18) and 2022 (Epi week 17) and extending to Epi weeks 40 and 51, respectively, for total durations of 22 and 34 weeks. Of note, an alternative analysis was conducted due to the very early disease onset post–COVID‐19 by redefining the RSV seasons as Epi weeks 1–52 for 2021 and 2022. This did not change the length of the RSV bronchiolitis epidemics at TGH/USF (data not shown).

RSV bronchiolitis epidemics tended to be slightly shorter at Duke (median 10.5 weeks) compared to SUNY (median 12.5 weeks) pre–COVID‐19. In the two RSV seasons following the onset of COVID‐19, the RSV bronchiolitis epidemics at Duke each lasted 14 weeks, while the epidemics at SUNY lasted 15 weeks in 2021–2022 and 8 weeks in 2022–2023. The shorter RSV bronchiolitis epidemic at SUNY could partially be attributed to a 4‐week period (Epi weeks 45–48) that accounted for 50% of the total cases in the SUNY system that season. The duration of RSV bronchiolitis epidemics at Renown ranged from 9 to 15 weeks pre–COVID‐19 with a median duration of 11.5 weeks. In the post–COVID‐19 RSV seasons, the epidemics were slightly shorter, each lasting 10 weeks despite the early onset.

### 3.5 Comparison of Infant Bronchiolitis and RSV Bronchiolitis Epidemics

To account for infants with bronchiolitis due to other viruses and/or those who did not undergo RSV testing, epidemics were defined using infants classified as having bronchiolitis and compared to the previously described RSV bronchiolitis epidemics. In the pre–COVID‐19 seasons, the onset of bronchiolitis epidemics occurred at a median 1–4 weeks prior to the onset of RSV bronchiolitis epidemics at each site (Figure S2). Additionally, the bronchiolitis epidemics ended a median 3.5–6.5 weeks after the RSV bronchiolitis epidemics. As a result, the total duration of bronchiolitis epidemics was longer than the RSV bronchiolitis epidemics. The median durations were as follows: TGH/USF 24.5 weeks, Duke and SUNY 18.5 weeks; Renown 20 weeks. Peaks in weekly bronchiolitis encounters occurred within 2 weeks of the RSV bronchiolitis peaks at each site during each pre–COVID‐19 season except at TGH in 2017–2018 and 2019–2020 (Figure S2). The rolling 8‐week bronchiolitis peaks at each site prior to COVID‐19 also closely mirrored the pattern of 8‐week RSV bronchiolitis peaks (Figure S1).

During the post–COVID‐19 seasons, the bronchiolitis epidemics began during the exact same week as the RSV bronchiolitis epidemics at all sites, with the only exceptions being Renown in 2021–2022 (onset 8 weeks prior to the RSV bronchiolitis epidemic) and Duke and SUNY in 2022–2023 (onset at each was 3 weeks prior to the RSV bronchiolitis epidemic). The bronchiolitis epidemics also lasted longer than RSV bronchiolitis epidemics at each site, ranging from 1 to 13 weeks longer in 2021–2022 and 4 to 9 weeks longer in 2022–2023 (Figure S2). Weekly bronchiolitis encounters peaked at the exact same time as RSV bronchiolitis encounters in each of the post–COVID‐19 seasons at Duke, SUNY, and Renown with the only exception being Renown in 2022–2023 (1 week difference). Weekly bronchiolitis encounters peaked at TGH/USF 5 and 4 weeks after RSV bronchiolitis peaks in 2021–2022 and 2022–2023, respectively (Figure S2).

## 4 Discussion

We used real‐time clinical and viral surveillance to examine the geographic progression of infant RSV bronchiolitis across the US during the COVID era. Despite PHSMs implemented during the COVID‐19 pandemic resulting in the near absence of infant RSV bronchiolitis after March 2020 with inter‐seasonal disease emerging in 2021 and early seasonal disease in 2022 [6, 9, 10, 11], RSV bronchiolitis epidemics progressed across the nation in a pattern identical to the prepandemic era, starting first in Florida and then spreading north and west across the US [7, 8, 10].

While our study does not indicate the optimal methodology for determining the start of the seasonal RSV bronchiolitis epidemic, it suggests that the geographic trend of RSV bronchiolitis epidemics across the US could be used to predict seasonal onset, as this pattern was unchanged even in the event of significant global disruption of viral transmission due to the implementation of PHSMs. Even though RSV epidemics started as early as late spring and summer in 2021–2022 and 2022–2023 (when the climate is very different than the typical time of epidemic onset in October–December), RSV bronchiolitis cases among infants progressed across these health care systems in the same pattern as prior to COVID‐19 onset. Although the cause for the preserved geographic progression of infant RSV bronchiolitis across the US is beyond the scope of this study, prior studies have shown latitudinal [12, 13, 14], longitudinal [15], and environmental [16] drivers of RSV epidemiology. It is also unknown if widespread implementation of maternal RSV immunizations and nirsevimab will disrupt this geographic progression, especially if there is variable uptake across health care systems.

Our results are consistent with the geographic pattern of RSV PCR tests across all ages post–COVID‐19 [10] but are strengthened by the inclusion of clinical criteria to specifically identify symptomatic, medically attended infants across all health care settings (inpatient, outpatient, ICU, and ED). In addition, our surveillance included both antigen and PCR tests to confirm RSV bronchiolitis. A limitation of our study was that it was not designed to assess or compare incidence rates of infant RSV bronchiolitis across these health care systems, which would depend on both regional sociodemographic factors and RSV testing practices within each health care system. The impact of COVID‐19 on RSV incidence has been previously described [6, 17], and it is unlikely that differences in RSV testing practices among each health care system affected our methodology as the geographic progression of infant bronchiolitis epidemics (with or without a confirmed RSV test) closely mirrored the geographic progression of infant RSV bronchiolitis epidemics (Figure S2). Regardless, further studies are underway to analyze the impact of COVID‐19 on RSV testing patterns among infants within the health care systems in our study (Blatt et al., under review).

The dynamics of US infant RSV bronchiolitis have nearly transitioned back to pre–COVID‐19 patterns; however, ongoing surveillance will be key to not only assess the impact of maternal RSV immunizations and nirsevimab on infant RSV bronchiolitis but also to determine whether these agents alter the geographic progression of disease across the US. Ultimately, understanding the geographic progression of RSV bronchiolitis among infants can augment surveillance data to help health care systems better understand precisely when they need to be optimally prepared for RSV bronchiolitis surges. Only with continued viral surveillance efforts, widespread implementation of preventative agents, and progress toward the development of new therapeutic options can the burden of this disease among infants be alleviated.

## Author Contributions

**Adam Z. Blatt:** Formal analysis; Visualization; Writing – original draft; Writing – review and editing. **Mina Suh:** Conceptualization; Data curation; Formal analysis; Methodology; Writing – review and editing. **Emmanuel B. Walter Jr:** Project administration; Supervision; Writing – review and editing. **Charles T. Wood:** Project administration; Supervision; Writing – review and editing. **Claudia Espinosa:** Project administration; Supervision; Writing – review and editing. **Maria E. Enriquez‐Bruce:** Project administration; Supervision; Writing – review and editing. **Joseph Domachowske:** Project administration; Supervision; Writing – review and editing. **Danielle Daniels:** Project administration; Supervision; Writing – review and editing. **Sonia Budhecha:** Project administration; Supervision; Writing – review and editing. **Amanda Elliott:** Project administration; Supervision; Writing – review and editing. **Zachary Wolf:** Conceptualization; Data curation; Formal analysis; Methodology; Supervision; Writing – review and editing. **Emory B. Waddell:** Conceptualization; Data curation; Project administration. **Naimisha Movva:** Conceptualization; Data curation; Methodology; Writing – review and editing. **Heidi Reichert:** Conceptualization; Data curation; Methodology. **Jon P. Fryzek:** Conceptualization; Data curation; Methodology; Writing – review and editing. **Christopher B. Nelson:** Conceptualization; Data curation; Formal analysis; Methodology; Project administration; Supervision; Writing – review and editing.

## Conflicts of Interest

C.B.N. is an employee of Sanofi. E.B.W has received funding support from Pfizer, Moderna, Sequiris, Najit Technologies Inc, and Clinetic for the conduct of clinical trials and clinical research. E.B.W. has served as an advisor to Vaxcyte and consultant to ILiAD biotechnologies. J.D. has received funding support from Pfizer, Moderna, Glaxo Smith Kline, Sanofi, Astra Zeneca, and Clinetic for the conduct of clinical trials and clinical research. J.D. has served as a consultant to Astra Zeneca, GlaxoSmithKline, and Moderna. C.E served as advisor for Sanofi and has received research support from Sanofi, AstraZeneca, and Clinetic. M.S., N.M., H.R., and J.P.F. are employees of EpidStrategies; EpidStrategies has received research funding from Sanofi, AstraZeneca, and Sobi.

### Peer Review

The peer review history for this article is available at https://www.webofscience.com/api/gateway/wos/peer‐review/10.1111/irv.13298.

## Supporting information


**Figure S1:** Geographic variation in the timing of bronchiolitis and RSV bronchiolitis peaks pre–COVID‐19.
**Figure S2:** Annual variation in medically attended bronchiolitis epidemics among infants at each health care site.

## Data Availability

Restrictions apply to the availability of these data, which are accessed pursuant to a contract between the health care systems and Clinetic.
