# Supplementary material for: Geographic Progression of Infant Respiratory Syncytial Virus Associated Bronchiolitis Across the United States Before and Since the Onset of COVID‐19: Results From Four Health Systems, 2015–2023
Source: Influenza Other Respir Viruses. 2024 May 15;18(5):e13298. doi: 10.1111/irv.13298 (PMC11096694; doi:10.1111/irv.13298)
Supplement: Supplementary file 1 — Figure S1: Geographic variation in the timing of bronchiolitis and RSV bronchiolitis peaks pre–COVID‐19. Figure S2: Annual variation in medically attended bronchiolitis epidemics among infants at each health care site. [file IRV-18-e13298-s001.docx]

**Supplementary Figure Legends:**

**Supplementary Figure 1: Geographic variation in the timing of bronchiolitis and RSV bronchiolitis peaks pre-COVID-19.** The weeks that comprised the greatest number of bronchiolitis and RSV bronchiolitis cases among infants <12 months old in a consecutive 8-week period at each site from 2015-2016 through 2019-2020 are plotted above. The width of the violin plot at each site represents the frequency that a particular Epi week was part of the worst 8-week period of cases, with the maximum number being 5 (representing each of the pre-Covid seasons). As an example, if a site had the exact same rolling 8-week peak every single season, then it would be represented by a perfect rectangle. The left panel represents bronchiolitis and the right panel represents RSV bronchiolitis. TGH/USF - Tampa General Hospital and University of South Florida Health in Tampa, FL; Duke - Duke University Health System in Durham, NC; SUNY - State University of New York Upstate Medical University Health System in Syracuse, NY; and Renown - Renown Regional Medical Center Health System in Reno, NV.

**Supplementary Figure 2: Annual variation in medically attended bronchiolitis epidemics among infants at each health care site.** Each bar represents contiguous Epi weeks that comprise 75% of a health care system’s total bronchiolitis encounters among infants <12 months old during a particular RSV season (defined as Epi week 27 through Epi week 26 of the subsequent year). The black dot represents the week within the epidemic that had the peak number of cases. Two bars are shown at TGH/USF for the 2021-2022 season due to the onset of RSV cases prior to Epi week 27 in 2021. TGH/USF - Tampa General Hospital and University of South Florida Health in Tampa, FL; Duke - Duke University Health System in Durham, NC; SUNY - State University of New York Upstate Medical University Health System in Syracuse, NY; and Renown - Renown Regional Medical Center Health System in Reno, NV.

**Supplementary Figures:**

**Supplementary Figure 1:**

**
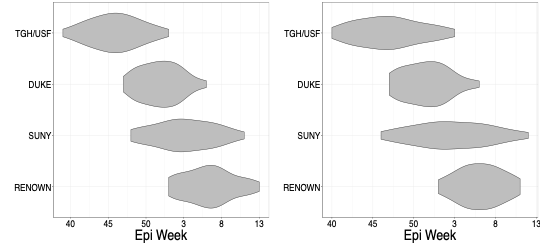
**

**Supplementary Figure 2:**

**
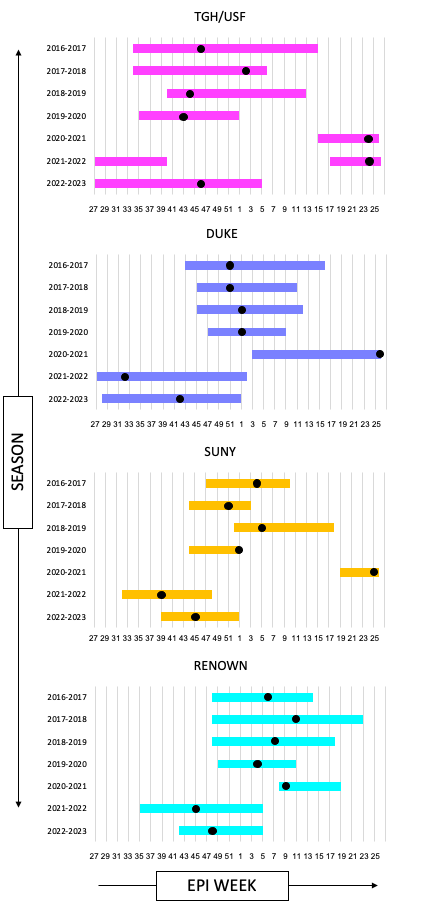
**
